# *Mycobacterium abscessus* Glycopeptidolipid Prevents Respiratory Epithelial TLR2 Signaling as Measured by HβD2 Gene Expression and IL-8 Release

**DOI:** 10.1371/journal.pone.0029148

**Published:** 2011-12-21

**Authors:** Lisa B. Davidson, Rachid Nessar, Prakasha Kempaiah, Douglas J. Perkins, Thomas F. Byrd

**Affiliations:** 1 Department of Medicine, New Mexico Veterans Health Care System, Albuquerque, New Mexico, United States of America; 2 Universite Paris V-Descartes, Faculté de Medecine, Paris, France; 3 The University of New Mexico School of Medicine, Center for Global Health, Albuquerque, New Mexico, United States of America; 4 The University of New Mexico School of Medicine, Division of Infectious Diseases, Albuquerque, New Mexico, United States of America; National Institute of Environmental Health Sciences, United States of America

## Abstract

*Mycobacterium abscessus* has emerged as an important cause of lung infection, particularly in patients with bronchiectasis. Innate immune responses must be highly effective at preventing infection with *M. abscessus* because it is a ubiquitous environmental saprophyte and normal hosts are not commonly infected. *M. abscessus* exists as either a glycopeptidolipid (GPL) expressing variant (smooth phenotype) in which GPL masks underlying bioactive cell wall lipids, or as a variant lacking GPL which is immunostimulatory and invasive in macrophage infection models. Respiratory epithelium has been increasingly recognized as playing an important role in the innate immune response to pulmonary pathogens. Respiratory epithelial cells express toll-like receptors (TLRs) which mediate the innate immune response to pulmonary pathogens. Both interleukin-8 (IL-8) and human β-defensin 2 (HβD2) are expressed by respiratory epithelial cells in response to toll-like receptor 2 (TLR2) receptor stimulation. In this study, we demonstrate that respiratory epithelial cells respond to *M. abscessus* variants lacking GPL with expression of IL-8 and HβD2. Furthermore, we demonstrate that this interaction is mediated through TLR2. Conversely, *M. abscessus* expressing GPL does not stimulate expression of IL-8 or HβD2 by respiratory epithelial cells which is consistent with “masking” of underlying bioactive cell wall lipids by GPL. Because GPL-expressing smooth variants are the predominant phenotype existing in the environment, this provides an explanation whereby initial *M. abscessus* colonization of abnormal lung airways escapes detection by the innate immune system.

## Introduction

*Mycobacterium abscessus*, a nontuberculous mycobacterium, is an important emerging pathogen causing fibrocavitary lung disease which is often indistinguishable from disease caused by *M. tuberculosis* [1]–[3]. It is also an emerging infection in patients with cystic fibrosis [4]–[7]. Unlike *M. tuberculosis*, *M. abscessus* has the ability to express glycopeptidolipid (GPL) in the outer cell wall. Expression of GPL, or lack thereof, is correlated with the smooth or rough colony phenotype respectively, which is observed in certain species of nontuberculous mycobacteria [8]. GPL-mediated biofilm formation is felt to facilitate survival in the environment and we have postulated that it may facilitate colonization of ectatic lung airways [8]. Spontaneous loss of GPL by *M. abscessus* is associated with acquisition of an invasive, immunostimulatory phenotype [8], [9]. We have proposed that loss of GPL “unmasks” underlying bioactive cell wall lipids which mediate virulence [8], [9]. Recently we deleted the gene *mmpL4b*, a gene coding for a critical enzyme in the biosynthetic GPL pathway [10], [11], from the well characterized *M. abscessus* GPL-expressing variant 390S [8], [9], [12]. Assessing the interaction of this deletion mutant with human monocyte-derived macrophages, we demonstrated that loss of GPL is sufficient to convert this *M. abscessus* variant to a phenotype which is able to replicate in these cells and stimulate their toll-like receptors (TLRs) [13].

*M. abscessus* primarily causes lung disease in individuals who are immunosuppressed, or who have abnormal lung airways. Because *M. abscessus* is ubiquitous in the environment, innate immune responses must be highly effective in preventing infection as normal hosts are uncommonly infected. TLRs recognize pathogen-associated molecular patterns and are the transducers of the innate immune response [14]. Mononuclear phagocytes have been extensively studied in terms of their TLR responses because they are actively involved in surveillance at the interface of the mucosal surfaces and the environment. Additionally, it has been recognized that respiratory epithelial cells lining the lung airways also play a critical role in surveillance and the innate immune response [15]. An important downstream effect of TLR signaling in respiratory epithelial cells is release of the chemokine interleukin-8 (IL-8) which recruits neutrophils from the circulation to the site(s) of TLR activation in the lung airways. As such, IL-8 release into cell supernates has been used as readout for TLR stimulation in experiments examining TLR responses of respiratory epithelial cells cultured *in vitro*. Human β-defensin (HβD2) is an antimicrobial peptide known to be upregulated in respiratory epithelial cells by TLR signaling [16]. In this study we demonstrate the utility of measuring HβD2 gene expression, as well as IL-8 release, as readouts of respiratory epithelial cell responses to *M. abscessus*. Using these assays, we demonstrate that respiratory epithelial cell TLRs do not recognize the colonizing phenotype of *M. abscessus* which expresses GPL, and that loss of GPL through targeted deletion of the *mmpLb4* gene converts *M. abscessus* to a phenotype which is recognized by toll-like receptor 2 (TLR2) on respiratory epithelial cells.

## Methods

### Bacteria

The isogenic *M. abscessus* 390S (smooth colony morphotype expressing GPL), 390R and 390V (rough colony morphotype lacking GPL expression) variants have been previously characterized [8], [9], [12], [17]. The *mmpL4b* gene deletion mutant derived from the 390S variant, and the *mmpL4b* complemented mutant have been described in a recent publication [13]. Bacteria were maintained as titrated frozen stocks stored at −70° C with intermittent passage for 3 days on Middlebrook 7H11 agar plates supplemented with Middlebrook OADC (BD), followed by flash freezing. To prepare single-cell frozen bacterial stocks for experiments, lawns of the different bacterial strains were plated on Middlebrook 7H11 OADC agar plates and incubated at 37° C. After 3 days, bacteria were harvested and placed into sterile Eppendorf tubes containing 1.0 mL sterile PBS and three glass beads. Tubes were pulse-vortexed 50 times, after which residual aggregates of bacteria were allowed to settle for 20 min. The top 500 µL of bacterial supernate was removed and the supernates from two to four tubes were pooled in 50 mL conical tubes. Bacteria were then sonicated on high power in a sonicating waterbath for 20 s to break apart any residual bacterial aggregates. The bacterial suspension was aliquoted into multiple Eppendorf tubes, which were then flash frozen and stored at −70° C. Individual tubes were thawed and titrated to determine CFU for a particular frozen stock.

### Infection of A549 cells for measurement of HBD2 gene expression and IL-8 levels

The A549 cell line is a well characterized Type II alveolar epithelial tumor cell line which was obtained from the American Type Culture Collection (Rockville, MD). A549 cells were cultured in Dulbecco's Modified Eagle Medium (DMEM) (Sigma) with 10% Fetal Bovine Serum (FBS)(Invitrogen) and 1% Antibiotic-Antimycotic (Gibco) at 37°C in 5% CO_2_. All experiments were performed prior to the twenty fifth cell passage. A549 cells were plated at 1.5×10^5^ cells/mL, in a volume of 3 mL of DMEM+10% FBS+1% Antibiotic-Antimycotic in 6-well tissue culture treated plates (Greiner) and incubated for 72 h at 37°C, in humidified 5% CO_2_. The cells were then washed 3 times with 3 mL Iscove's Modified Dulbecco's Medium (with sodium bicarbonate; without L glutamine: Sigma), and the media was replaced with 2 mL Iscoves+5% human serum (Human AB Serum, male—SeraCare). Wells were infected with *M. abscessus* variants at a concentration of 1×10^7^ CFU/well. Uninfected control wells were left untreated or in some cases received either 100 ng/mL MALP-2 (TLR6/TLR2 ligand; Imgenex), or 20 ng/mL recombinant human interleukin-1β (IL1ß) (PeproTech). The plates were then incubated at 37°C, in humidified 5% CO_2_ for 8 hr. The wells were washed 3 times with 3 mL Iscove's medium, and 350 µL Qiagen RLT buffer (Qiagen) was added to the wells to lyse the cells. Lysates were placed in RNAse free tubes (VWR) and frozen −80°C for later RNA isolation.

For experiments measuring A549 IL-8 release, cells were plated at a concentration of 5×10^5^ cells/mL using the same medium in 24-well tissue culture treated plates (Greiner), and incubated for 24 h at 37°C, in humidified 5% CO_2_. The cells were then washed 3 times with 0.5 mL Iscoves medium and the media replaced with Iscoves medium alone. Wells then received medium alone, anti-TLR2 antibody (10 µg/mL)(Ebioscience 16902483) or IgG_1_ isotype control antibody (10 µg/mL)(Ebioscience 16902483). After a 1 h incubation, wells received no bacteria, or *M. abscessus* variants at a concentration of 2.5×10^6^ CFU/well. After an additional 8 hours the supernates were collected, filtered with 0.2 um centrifugal filters (VWR 82031-358), and frozen at −80°C. Supernates were analyzed per manufacturer's protocol with the BD Human IL-8 ELISA set (BD555244).

### Infection of BEAS 2B cells for measurement of IL-8 levels

The BEAS 2B cell line, a well-characterized SV40 immortalized cell line derived from normal human bronchial epithelium, was obtained from the ATCC. These cells contain TLR1 through TLR6 and respond to the TLR2 agonist Pam 3 Cys with expression of IL-8 [18], [19]. BEAS2b cells were plated at a concentration of 2×10^5^ cells per well in 0.5 mL BEBM+BEGM kit (without gentamicin-amphotericin B) (Lonza cc3171 and cc3170), in 24-well tissue culture plates coated with 0.01 mg/mL fibronectin, 0.3 mg/mL bovine collagen type 1 and 0.01 mg/mL BSA in BEBM (Sigma F1141; Advanced Biomatrix 5005-B; Sigma A9418), and incubated at 37°C, 5% CO_2_ for 24 hours. The wells were rinsed 3 times with BEBM and refilled with 0.5 mL BEBM. Wells then received medium alone, anti-TLR2 antibody (10 µg/mL)(Ebioscience 16902483) or IgG_1_ isotype control antibody (10 µg/mL)(Ebioscience 16902483). Wells were then incubated at 37°C, 5% CO_2_ for 1 hour. After 1 h incubation, wells received no bacteria, or *M. abscessus* variants at a concentration of 2.5×10^6^ CFU/well. After an additional 8 hours, the supernates were collected and filtered with 0.2 um centrifugal filters (VWR 82031-358) and frozen at −80°C. Supernates were analyzed per manufacturers protocol with the BD Human IL-8 ELISA set (BD555244).

### Real-time PCR assessment of HBD2 gene expression

RNA from lysates of A549 cells receiving various treatments was isolated using the RNeasy kit (Qiagen). For HβD2, the primer/probe sequences and final reaction concentrations were based on previous reports, and were as follows: forward: 5′-GAGGAGGCCAAGAAGCTGC-3′ (300 nM); reverse: 5′-CGCACGTCTCTGATGAGGG-3′ (300 nM); probe: 5′-FAM-TGGCTGATGCGGATTCAGAAAGGG-TAMRA-3′ (250 nM) [16]. The ABI Human Eukaryotic 18S rRNA Taqman® Gene Expression Assay (Endogenous Control) was used per the manufacturer's instruction for detection of 18S rRNA. qRTPCR of the RNA was performed using the ABI Taqman® One Step RT-PCR Master Mix Reagents kit, per manufacturer's instruction. A no template control was included to verify that amplification only took place in reactions containing RNA. Thermocycling conditions began with reverse transcription, consisting of one cycle at 48°C for 30 minutes, followed by one cycle at 95°C for 10 minutes. PCR consisted of 40 cycles at 95°C for 15 seconds, and 60°C for 60 seconds. Relative quantity was determined using the 2^−ΔΔC^T method [20], using the untreated control as the calibrator sample.

### Assessment of the effect of siRNA TLR2 knockdown on HBD2 expression in response to M. abscessus

Transfection of A549 cells was performed by plating at a concentration of 4×10^5^ cells per well in 4 mL DMEM+10% FBS +1% Antibiotic-Antimycotic in 6 well tissue culture plates followed by incubation at 37°C in humidified 5% CO_2_ for 24 hours. The cells were then washed 3 times with 2 mL 37° C PBS (Gibco), followed by addition of 2 mL 37° C DMEM+10% FBS+1% Antibiotic-Antimycotic to each well. The transfection solution was created by combining 2.2 pmoles of either TLR2 Silencer Select siRNA s169 (ABI) or Silencer Select Negative Control #1 siRNA (ABI) to knock down TLR2 expression and serve as a negative control respectively, 8 µL INTERFERin (Polyplus) and 200 µL DMEM. After a 10 minute incubation period at room temperature, 200 µL of the transfection solution was added to each well of a 6 well plate containing A549 cells, and 200 µL DMEM was added to untreated control wells. Plates were then incubated at 37°C, in humidified 5% CO_2_ for 48 hours. Tissue culture plates containing transfected A549 cells were washed 3 times with 2 mL Iscove's medium, and *M. abscessus* 390S Δ*mmpL4b* was added to wells at a concentration of 1×10^7^ CFU/well, in 2 mL Iscove's+5% human serum. Control wells received only 2 mL Iscove's medium+5% human serum. Plates were then incubated at 37°C, in humidified 5% CO_2_ for 8 hours. The wells were then washed three times with Iscove's medium, prior to being lysed with 350 µL Qiagen RLT buffer (Qiagen). Lysates were placed in RNAse free tubes (VWR) and frozen −80°C for later RNA isolation.

RNA was isolated using the RNeasy kit. The HβD2 primer/probe sequences (see above), the ABI TLR2 gene expression assay Hs00152932_m1, and the ABI Human Eukaryotic 18 s rRNA Taqman Gene Expression Assay (Endogenous Control) were used for detection of HβD2, TLR2 and 18 s, respectively. qRTPCR of the RNA was performed as described above. A no template control was included to verify that amplification only took place in reactions containing RNA. Thermocycling conditions were as described above. Relative quantity was determined using the 2^−ΔΔC^T method [20], using the untreated control as the calibrator sample for HβD2 expression and the control treated with scrambled RNA as the control sample for TLR2 expression.

### Western blot analysis

Transfected A549 cells were lysed with 300 µL RIPA buffer (Pierce). Lysates were placed in sterile tubes (VWR), and frozen −80°C for later analysis. Protein concentration was measured using the BCA protein Assay (Thermo). 50 µg protein was incubated in 1× Laemmli Sample Buffer (Biorad) at 95°C for 5 minutes, and separated by electrophoresis on a 10% Tris-HCL ReadyGel (Biorad) in Tris/Glycine/SDS Buffer (Biorad). The protein was then transferred to a 0.2 µm nitrocellulose membrane (Biorad) in 10× Tris/Glycine Buffer (Biorad)+20% methanol (Fisher), overnight at 4°C. The membrane was blocked with 5% nonfat dry milk in PBS-0.05% tween (PBS, Gibco; Tween-20, Sigma), for one hour at room temperature. Following 3 washes in PBS-0.05% tween, the membrane was cut such that the actin bands were on one half and the TLR2 bands on the other. The TLR2 membrane was incubated in 2 µg/mL Rabbit polyclonal antibody toTLR2 (Abcam), and the actin membrane in 0.5 µg/mL mouse monoclonal antibody to actin (Abcam), in 3% BSA- PBS-0.05% Tween (BSA, EM Science), for one hour at room temperature. Following 3 washes in PBS-0.05% Tween, the TLR2 membrane was incubated in 1∶250,000 goat anti-rabbit IgG+HRP (Thermo), and the actin membrane in 1∶250,000 goat anti-mouse IgG+HRP (Thermo), in 5% nonfat dry milk in PBS-0.05% tween, for one hour at room temperature. After 3 washes in PBS-0.05% Tween, the membranes were blotted dry on bibulous paper before 1 minute of incubation with Supersignal West Dura Extended Duration Substrate (Thermo). The membranes were dried with bibulous paper, and exposed to blue x-ray film (Phoenix).

## Results

### A549 alveolar epithelial cells generate an innate immune response to M. abscessus variants lacking GPL

**Figure 1:**
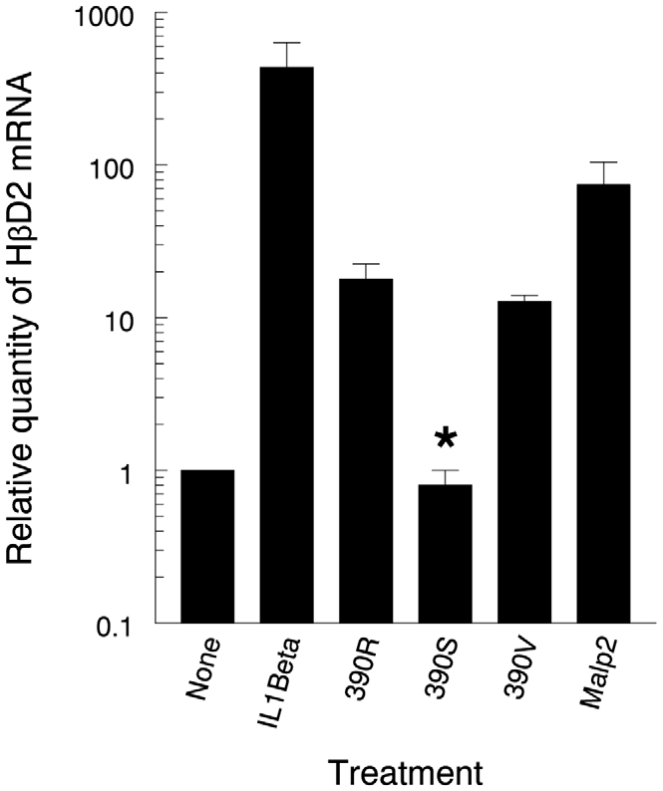
A549 cells increase HβD2 gene expression in response to *M. abscessus* variants lacking GPL, but not the *M. abscessus* 390S variant expressing GPL. A549 cell monolayers were uninfected or challenged with *M. abscessus* variants 390R or 390S. In addition, some uninfected A549 cell monolayers were treated with IL1β or MALP-2 as controls for the ability of A549 cells to upregulate HβD2 gene expression. After 8 hours, HβD2 gene expression was quantified by real-time PCR. The results of real-time PCR are expressed as the relative fold increase in HBD2 gene expression over that of the untreated group and presented as mean +/− SD of measurements from the same experiment performed in triplicate. * 390S versus 390R and 390V; *P*<0.05, *t*-test.

We have previously demonstrated that human macrophages recognize *M. abscessus* variants lacking GPL via TLR2, resulting in release of the proinflammatory cytokine TNFα. One class of *M. abscessus* surface molecules involved in this interaction are the phosphatidyl-*myo*-inositol mannosides which we have demonstrated are “masked” in *M. abscessus* variants expressing GPL [9]. To determine whether the innate immune system of respiratory epithelial cells recognizes *M. abscessus* rough variants lacking GPL, we challenged A549 cells with the *M. abscessus* rough variants 390R and 390V, and the *M. abscessus* smooth variant 390S which expresses GPL [8], [9]. HβD2, which is expressed by A549 cells in response to TLR stimulation [18], was assessed by real-time PCR. IL-1β stimulation without *M. abscessus* infection was included as one control because it stimulates HβD2 expression by a signaling pathway which is independent from the TLR signaling pathway [16]. MALP-2 stimulation without *M. abscessus* infection was included as another control because it signals through the TLR2 signaling pathway [21]. Both IL-1β and MALP-2 stimulation resulted in substantial increases in HβD2 gene expression relative to untreated controls. Both *M. abscessus* rough variants lacking GPL also stimulated a significant increase in HβD2 gene expression over untreated control and *M. abscessus* 390S. In contrast, *M. abscessus* 390S did not increase HβD2 gene expression above untreated control (Figure 1). These results indicate that *M. abscessus* rough variants stimulate the innate immune response of respiratory epithelial cells.

### A M. abscessus GPL deletion mutant derived from the 390S smooth variant regains the ability to stimulate the A549 alveolar epithelial cell innate immune response

**Figure 2:**
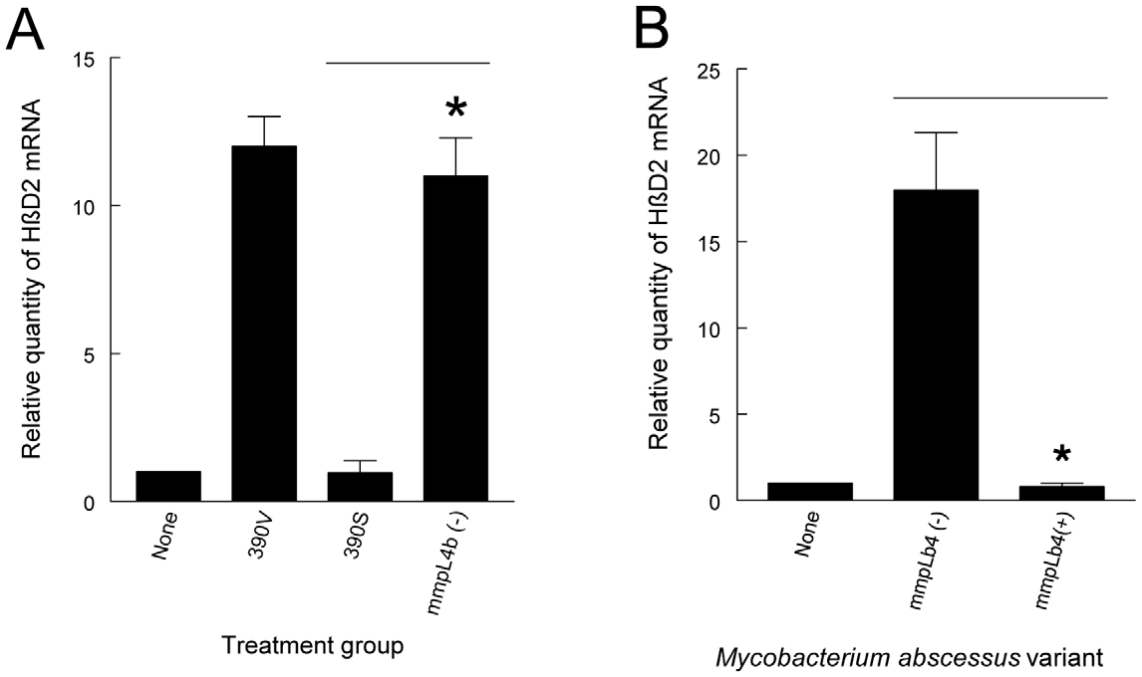
A *M. abscessus* 390SΔ*mmpL4b* deletion mutant lacking GPL has acquired the ability to stimulate HBD2 gene expression in A549 cells. (A) A549 cell monolayers were uninfected or challenged with *M. abscessus* variants 390V, 390S or 390SΔ*mmpL4b*, a deletion mutant lacking the *mmpL4b* gene which is a critical component of the GPL biosynthetic pathway. The results of real-time PCR are expressed as the relative fold increase in HβD2 gene expression over that of the untreated group and presented as mean +/− SD of measurements from the same experiment performed in triplicate. * 390SΔ*mmpL4b* mutant vs 390S wild type; *P*<0.05, *t*-test. (B) A549 cell monolayers were uninfected or challenged with *M. abscessus* 390SΔ*mmpL4b*, or the complemented 390SΔ*mmpL4b* mutant. After 8 hours, HβD2 gene expression was quantified by real-time PCR. The results of real-time PCR are expressed as the relative fold increase in HβD2 gene expression over that of the untreated group and presented as mean +/− SD of measurements from the same experiment performed in triplicate. * 390SΔ*mmpL4b* complemented versus 390SΔ*mmpL4b* mutant; *P*<0.05, *t*-test.

We next sought to determine whether the previously characterized deletion mutant 390SΔ*mmpL4b*, which does not express GPL [13], gains the ability to stimulate the innate immune response of respiratory epithelial cells. When challenged with the 390SΔ*mmpL4b* variant, A549 cells responded with increased expression of HβD2 to a level comparable to that seen with the rough variant 390V (Figure 2A). *M. abscessus* 390V is the ideal comparator strain in this assay because it is a spontaneous mutant which arose on subculture of 390S, acquiring the rough phenotype and losing its ability to express significant quantities of GPL [8]. In addition, the complemented 390SΔ*mmpL4b* strain, which has regained the smooth phenotype and the ability to produce GPL [13], lacks the ability to stimulate A549 cell HβD2 gene expression (Figure 2B). Taken together, these results indicate that *M. abscessus* GPL expression interferes with activation of the respiratory epithelial cell innate immune response.

### TLR2 siRNA treatment decreases expression of TLR2 in both uninfected and infected A549 alveolar epithelial cells

**Figure 3:**
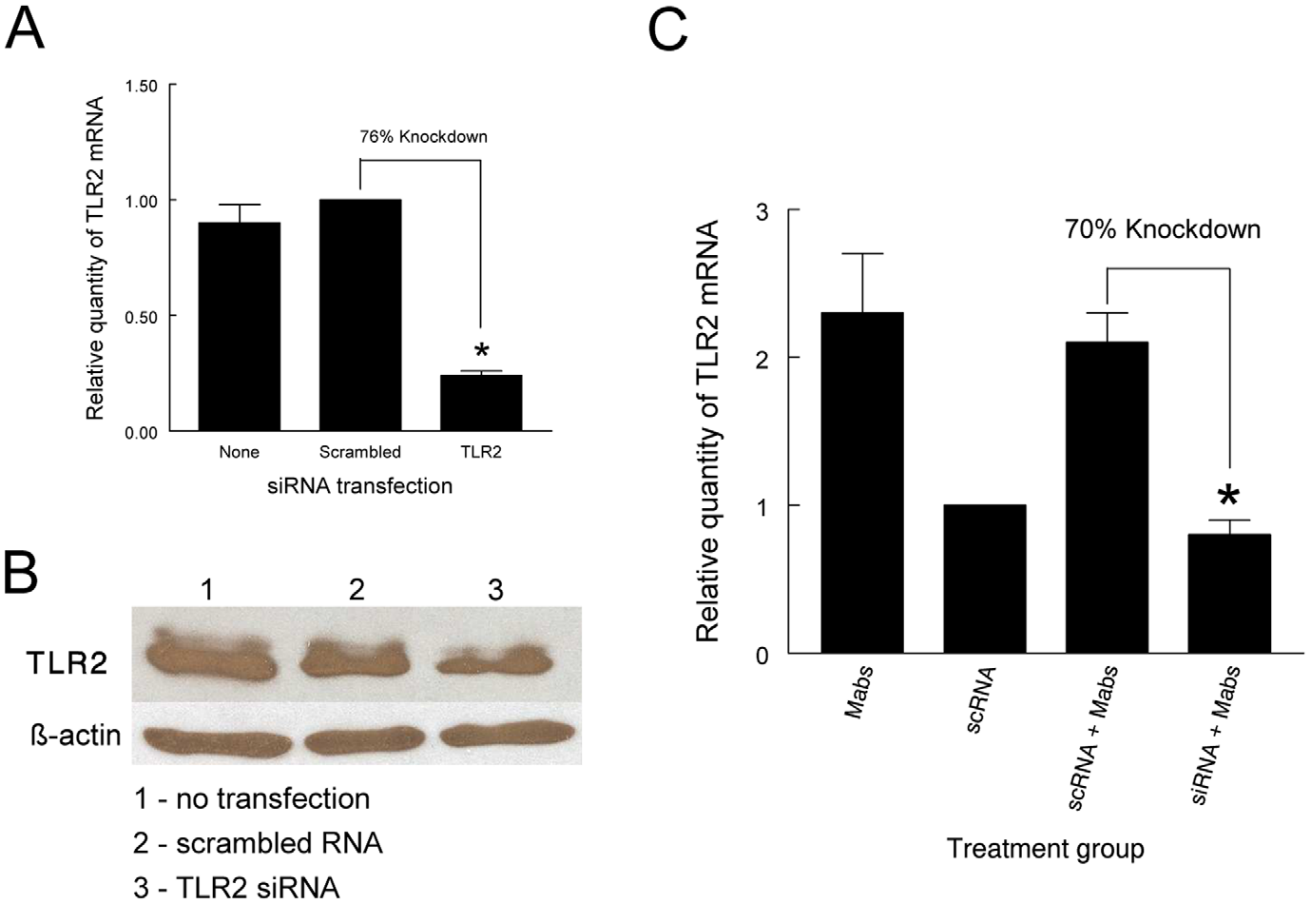
TLR2 siRNA treatment decreases TLR2 gene expression in uninfected and *M. abscessus* -infected A549 cells. As a first step in assessing the role of TLR2 in respiratory epithelial responses to *M. abscessus*, the effect of transfection of A549 cells with TLR2 siRNA on TLR2 gene expression was assessed. (A) Uninfected A549 cells were transfected with scrambled RNA or TLR2 siRNA. After 48 h TLR2 gene expression was quantified by real-time PCR. The results of real-time PCR are expressed as the relative difference in TLR2 gene expression using the A549 monolayers receiving scrambled RNA as the reference, with data presented as mean +/− SD of measurements from the same experiment performed in triplicate. *TLR2 transfected cells versus cells receiving scrambled RNA *P*<0.05, *t*-test. (B) Western blotting of A549 cell extracts from (A) demonstrates decreased TLR2 in cells treated with TLR2 siRNA. (C) A549 cell monolayers were either untreated or transfected with RNA, and either uninfected or challenged with *M. abscessus* 390SΔ*mmpL4b*. TLR2 gene expression was quantified by real-time PCR. The results of real-time PCR are expressed as the relative difference in TLR2 gene expression using uninfected A549 monolayers receiving scrambled RNA as the reference, with data presented as mean +/− SD of measurements from the same experiment performed in triplicate. * TLR2 siRNA **+** *M. abscessus* 390SΔ*mmpL4b* versus scrambled RNA + *M. abscessus* 390SΔ*mmpL4b*; *P*<0.05, *t*-test.

In a prior study we demonstrated that human macrophage TLR2 mediates the macrophage innate immune response to *M. abscessus* rough variants [9]. As the first step in assessing the role of TLR2 in the respiratory epithelial response to *M. abscessus*, we examined the effect of TLR2 siRNA on TLR2 gene expression in uninfected A549 cells. TLR2 siRNA treatment resulted in a 76% reduction in TLR2 gene transcript relative to cells treated with scrambled RNA (Figure 3A). This was accompanied by a decrease in TLR2 protein as assessed by Western blotting (Figure 3B). When A549 cells were infected with *M. abscessus* 390SΔ*mmpL4b*, baseline expression of TLR2 increased. TLR2 siRNA treatment, however, resulted in decreased TLR2 gene expression, while treatment with scrambled RNA did not (Figure 3C). These results indicate that siRNA TLR2 treatment is able to reduce TLR2 gene expression in both uninfected and infected A549 cells.

### TLR2 siRNA treatment decreases expression of HβD2 gene transcript in A549 alveolar epithelial cells in response to challenge with the M. abscessus 390SΔmmpL4b deletion mutant

**Figure 4:**
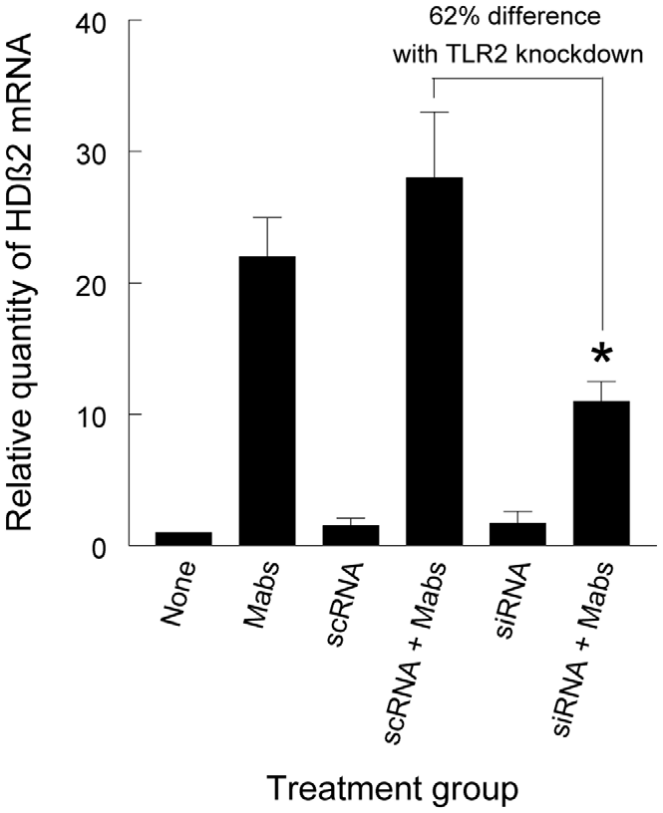
TLR2 siRNA treatment decreases HβD2 gene expression in A549 cells challenged with the *M. abscessus* 390S *mmpL4b* deletion mutant. A549 cells were transfected with scrambled RNA or TLR2 siRNA for 48 h with some cell monolayers then challenged with *M. abscessus* 390SΔ*mmpL4b*. After 8 h, HβD2 gene expression was quantified by real-time PCR. The results of real-time PCR are expressed as the relative change in HβD2 gene expression over that of the untreated, uninfected group and presented as mean +/− SD of measurements from the same experiment performed in triplicate. * TLR2 siRNA **+** *M. abscessus* 390SΔ*mmpL4b* versus scrambled RNA + *M. abscessus* 390SΔ*mmpL4b*; *P*<0.05, *t*-test.

To determine whether the *M. abscessus* 390S*ΔmmpL4b* deletion mutant signals the innate immune response of A549 cells through TLR2, A549 cells were treated with scrambled RNA or TLR2 siRNA and then infected with this variant. Treatment with TLR2 siRNA, but not scrambled RNA, was associated with a significant reduction in HβD2 gene expression compared with cells receiving scrambled RNA (Figure 4). These results indicate that *M. abscessus* variants lacking GPL signal the respiratory epithelial cell response through TLR2.

### IL-8 release by A549 alveolar epithelial cells in response to the M. abscessus 390SΔmmpL4b deletion mutant is mediated by TLR2

**Figure 5:**
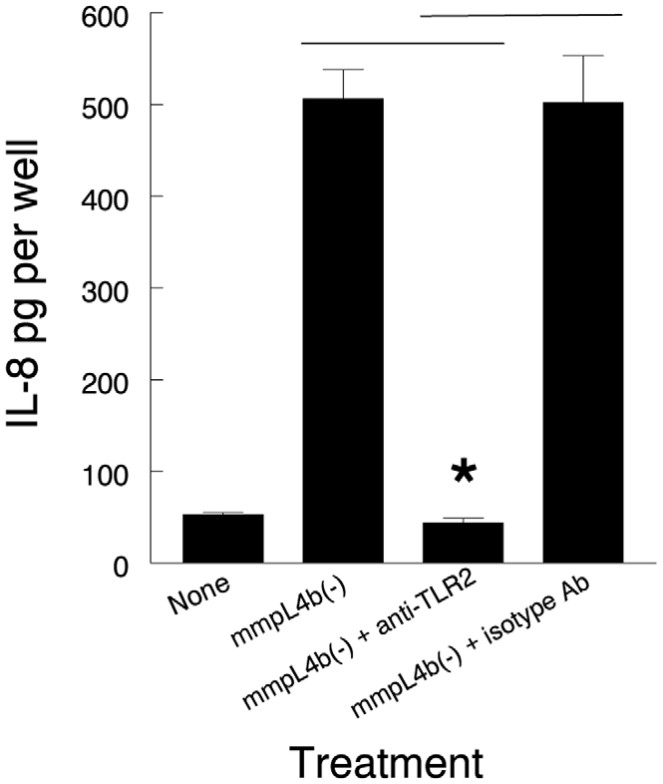
Antibody to TLR2 decreases IL-8 release from A549 cells in response to the *M. abscessus* 390SΔ*mmpL4b* deletion mutant. A549 cell monolayers were preincubated with antibody to TLR2 or isotype control antibody and then received no bacteria or were challenged with the *M. abscessus* 390SΔ*mmpL4b* deletion mutant. Culture supernates were collected after 8 h and assayed by ELISA for IL-8. Data are means ± SEM of two experiments done in triplicate. * 390SΔ*mmpL4b* + anti-TLR2 antibody versus 390SΔ*mmpL4b* alone and 390SΔ*mmpL4b* + isotype antibody; p<0.01, *t*-test.

IL-8 has been used as a readout for TLR2 stimulation of respiratory epithelial cells. To replicate the HβD2 experiments using a different method, we assessed the response of A549 cells to *M. abscessus* 390SΔ*mmpL4b* by measuring IL-8 release. In addition, we assessed the role of TLR2 in mediating this response using a TLR2 blocking antibody which we have previously used to block the human macrophage TLR2 response to *M. abscessus* variants [9]. In this experiment 390SΔ*mmpL4b* stimulated release of substantial quantities of IL-8 which was blocked by preincubation with anti-TLR2 antibody, but not isotype control antibody (Figure 5). These results are consistent with the results obtained using HβD2 gene expression as a readout for stimulation of TLR2 by *M. abscessus* 390SΔ*mmpL4b*.

### GPL expression by M. abscessus 390S prevents TLR2 signaling in BEAS 2B cells as measured by IL-8 release

**Figure 6:**
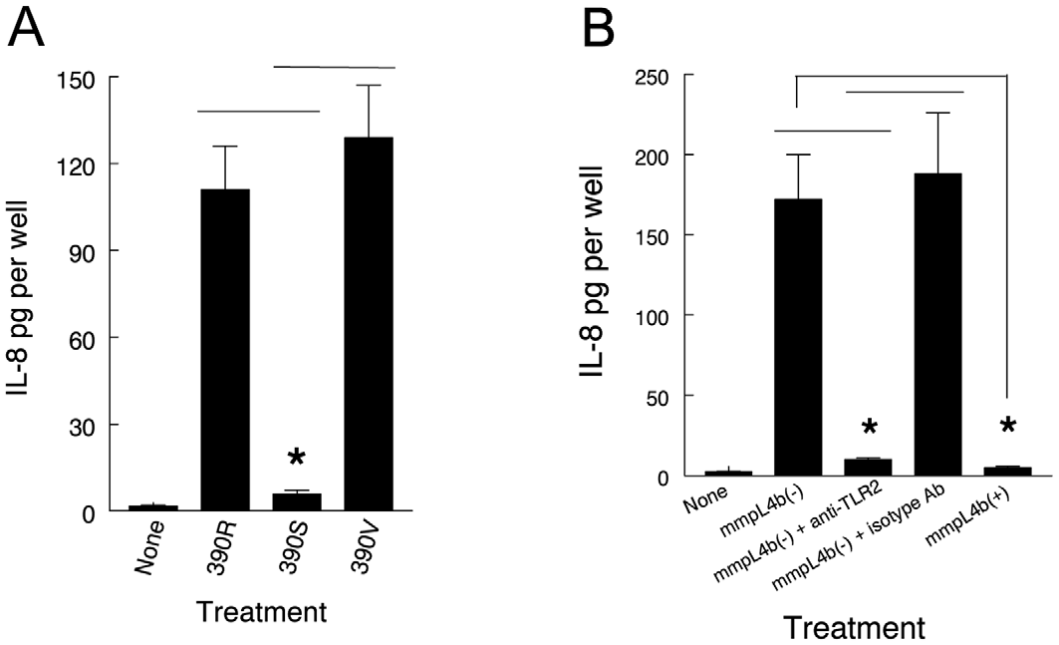
IL-8 release from BEAS 2B cells in response to *M. abscessus* variants lacking GPL is mediated by TLR2. (A) BEAS 2B bronchial epithelial cells received no bacteria or were challenged with *M. abscessus* variants 390R, 390S and 390V. Culture supernates were collected after 8 h and assayed by ELISA for IL-8. Data are means ± SEM of two experiments done in triplicate. * 390S versus 390R and 390V; p<0.01, *t*-test. (B) BEAS 2B bronchial epithelial cells were preincubated with no antibody, antibody to TLR2 or isotype control antibody. Monolayers then received no bacteria or were challenged with the *M. abscessus* 390SΔ*mmpL4b* deletion mutant or the 390SΔ*mmpL4b* complemented mutant. Culture supernates were collected after 8 h and assayed by ELISA for IL-8. Data are means ± SEM of two experiments done in triplicate. * 390SΔ*mmpL4b* deletion mutant + anti-TLR2 antibody versus 390SΔ*mmpL4b* deletion mutant alone and versus 390SΔ*mmpL4b* deletion mutant + isotype antibody; p<0.01. * 390SΔ*mmpL4b* complemented mutant versus 390SΔ*mmpL4b* deletion mutant; p<0.01.

To insure that our findings with A549 cells have relevance to other respiratory epithelial cells, we assessed the interaction of *M. abscessus* variants with BEAS 2B cells. These cells are a well-characterized SV40 immortalized cell line derived from normal human bronchial epithelium. They possess TLR2 receptors and release IL-8 in response to TLR2 stimulation [18], [19]. In these experiments we measured IL-8 release in response to our *M. abscessus* rough variants 390R and 390V, and the smooth variant 390S which expresses GPL. Both *M. abscessus* rough variants lacking GPL stimulated a significant increase in IL-8 release compared to the untreated control and *M. abscessus* 390S (Figure 6A). Furthermore, *M. abscessus* 390SΔ*mmpL4* regained the ability to stimulate IL-8 release via TLR2 (Figure 6B). These results indicate that *M. abscessus* variants lacking GPL either through spontaneous mutation, or targeted deletion of a gene critical for GPL formation, gain the ability to stimulate TLR2 in respiratory epithelial cells. These results are consistent with our previous findings which indicate that GPL masks underlying bioactive cell wall molecules capable of interacting with host cells [9].

## Discussion

In this study we demonstrate that a naturally occurring GPL-expressing smooth *M. abscessus* variant (390S) does not stimulate the innate immune response of respiratory epithelial cells, while rough variants lacking GPL (390R, 390V) stimulate respiratory epithelial cells through TLR2, resulting in gene expression of the downstream effector molecule HβD2 and release of IL-8. Furthermore, loss of *M. abscessus* 390S GPL through targeted deletion of the *mmpL4b* gene critical for GPL synthesis [13] converts the bacterium to a phenotype capable of stimulating respiratory epithelial TLR2. These results are in keeping with our prior studies using macrophages, which demonstrate that loss of GPL through either spontaneous mutation, physical removal, or targeted gene deletion converts *M. abscessus* to an immunostimulatory phenotype capable of stimulating release of TNFα via interaction with TLR2 [9], [13]. In previous studies we have provided evidence that GPL masks underlying *M. abscessus* cell wall lipids which are known TLR2 ligands [9].

There has been increasing recognition of the role that respiratory epithelium plays in host innate immune responses to bacterial pathogens. The human innate immune response is the immediate response engendered by the host to a foreign antigen. In the lung, both alveolar macrophages, and respiratory epithelial cells are central to the innate immune response [15]. It is distinct from adaptive or cell-mediated immunity which takes time to develop and involves antigen presentation to T- lymphocytes, which orchestrate the subsequent host response. Because *M. abscessus* is primarily an opportunistic pathogen, it is probable that innate immune responses are important in preventing infection with this organism. The TLRs, which mediate the host innate immune response, are present on macrophages, dendritic cells and respiratory epithelial cells lining the lung. These receptors recognize pathogen-associated molecular patterns, which are conserved motifs expressed by microorganisms, but not by higher eukaryotes. In the case of mycobacteria, bacterial lipopeptides in the cell wall are recognized by host cell TLR2/TLR1 heterodimers [22], [23]. We have previously demonstrated that *M. abscessus* expresses one type of these surface components, the phosphatidyl-*myo*-inositol mannosides [9], which stimulate the macrophage innate immune response via TLR2 signaling.

Engagement of TLRs on macrophages leads to gene expression of pro-inflammatory cytokines [23]. Ligand binding to TLRs present on respiratory epithelial cells leads to expression of the chemokine IL-8 involved in recruitment of circulating neutrophils to sites of infection/inflammation in the lung [24]. Stimulation of respiratory epithelial cells with the inflammatory cytokine IL-1β also leads to release of IL-8 through a mechanism that bypasses the TLR signaling [25]. In addition to IL-8, respiratory epithelial cells upregulate expression of the antimicrobial peptide HβD2 both in response to TLR engagement and IL1β stimulation [26]. Thus, there are two mechanisms by which respiratory epithelial cells are stimulated to express HβD2 and IL-8 in response to bacterial pathogens. In one mechanism, alveolar macrophages present in lung alveoli recognize bacteria via TLRs leading to expression of IL-1β. This in turn activates respiratory epithelial cells in the immediate vicinity to upregulate expression of HβD2 and release IL-8. This mechanism does not involve binding TLRs on respiratory epithelial cells [27]. In the second mechanism, direct engagement of TLRs at various levels in the respiratory tract by bacterial ligands results in a signaling cascade leading to HβD2 expression by respiratory epithelial cells [26] and release of IL-8. Thus, in upper portions of the respiratory tract where mucociliary clearance is operating and alveolar macrophages are absent, respiratory epithelial cells are the primary sentinels, sampling the airways for bacterial pathogens via TLRs on their surface. If bacteria are able to survive the upper airways and enter the alveoli, respiratory epithelial HβD2 expression and IL-8 release in response to direct TLR2 stimulation is augmented by IL-1β released from alveolar macrophages. In addition to inducing HβD2 by alveolar epithelial cells, IL-1β stimulates cell-mediated immune responses such as activation of B and T lymphocytes. This sequence of events is consistent with a vigorous host defense being mounted in the alveoli if upper airway defense mechanisms are breached.

A549 cells are an alveolar epithelial tumor cell line used to study respiratory epithelial cellular responses [28]. Because freshly explanted human alveolar type II cells are difficult to obtain, A549 cells are a useful surrogate for the study of respiratory epithelial cell responses at the level of the alveolus [29]. Of relevance to our study is the fact that these cells have been used to study the interaction of mycobacteria with respiratory epithelium [30]–[34]. Also relevant to our study are previous reports indicating that A549 cells express TLR2, express HβD2 in response to IL1-β and TLR2 stimulation [16], and express HβD2 in response to *M. tuberculosis* infection [35]. In addition, evidence suggests that HβD2 possesses antimicrobial activity against *M. tuberculosis* when expressed by host cells coming into contact with this bacterium [36]. In addition to A549 cells, we examined the interaction of *M. abscessus* variants with bronchial epithelial BEAS 2B cells. This was done to assess responses to *M. abscessus* by a respiratory epithelial cell from a different site of origin (bronchus) than A459 cells. We also wanted to replicate our findings in a non-tumor cell line because genetic changes associated with tumorigenesis can alter cellular responses. We found that both cell lines responded similarly to *M. abscessus* variants.

Our study demonstrates that *M. abscessus* GPL-expressing smooth variants are not recognized by TLRs present on respiratory epithelial cells. This is consistent with the hypothesis we have put forth which suggests that *M. abscessus* smooth variants are a colonizing phenotype by virtue of characteristics which include sliding motility, biofilm formation, and the ability to avoid detection by TLR2 [8], [9], [12], [17]. These features enable *M. abscessus* smooth variants, which are likely the predominant phenotype found in the environment [37], to colonize ectatic airways and escape recognition by the innate immune system. We have previously demonstrated that spontaneous loss of GPL converts *M. abscessus* to an immunostimulatory phenotype capable of invading and replicating in fibroblasts and macrophages [8], [9], [12], [13], [17]. We now extend our observations by demonstrating that *M. abscessus* variants lacking GPL are recognized by TLR2 on respiratory epithelial cells resulting in release of IL-8 and expression of HβD2. Based on these results and those of prior studies, we propose that *M. abscessus* variants expressing GPL are able to colonize abnormal upper airways in patients with bronchiectasis without evoking an immune response. This enables the bacteria to establish a foothold in the lung. Spontaneous mutants lacking GPL, arising from these colonizing bacteria, then enter the alveolar space where they cause invasive lung infection and provoke an inflammatory response. It is possible that additional defects in the immune recognition and/or response are present in subsets of patients with bronchiectasis which result in an inability to clear these rough variants once invasive infection is established in the lung parenchyma. This is currently being investigated in our laboratory.

## References

[1] Howard ST, Byrd TF (2000). The rapidly growing mycobacteria: saprophytes and parasites.. Microbes Infect.

[2] De Groote MA, Huitt G (2006). Infections due to rapidly growing mycobacteria.. Clin Infect Dis.

[3] Griffith DE, Girard WM, Wallace RJ (1993). Clinical features of pulmonary disease caused by rapidly growing mycobacteria. An analysis of 154 patients.. Am Rev Respir Dis.

[4] Fauroux B, Delaisi B, Clement A, Saizou C, Moissenet D (1997). Mycobacterial lung disease in cystic fibrosis: a prospective study.. Pediatr Infect Dis J.

[5] Cullen AR, Cannon CL, Mark EJ, Colin AA (2000). *Mycobacterium abscessus* infection in cystic fibrosis. Colonization or infection?. Am J Resp Crit Care.

[6] Olivier KN, Weber DJ, Wallace RJ, Faiz AR, Lee JH (2003). Nontuberculous mycobacteria. I: multicenter prevalence study in cystic fibrosis.. Am J Resp Crit Care.

[7] Sermet-Gaudelus I, Le Bourgeois M, Pierre-Audigier C, Offredo C, Guillemot D (2003). *Mycobacterium abscessus* and children with cystic fibrosis.. Emerg Infect Dis.

[8] Howard ST, Rhoades E, Recht J, Pang X, Alsup A (2006). Spontaneous reversion of *Mycobacterium abscessus* from a smooth to a rough morphotype is associated with reduced expression of glycopeptidolipid and reacquisition of an invasive phenotype.. Microbiology.

[9] Rhoades ER, Archambault AS, Greendyke R, Hsu F, Streeter C (2009). *Mycobacterium abscessus* glycopeptidolipids mask underlying cell wall phosphatidyl-myo-inositol mannosides blocking induction of human macrophage TNF-α by preventing interaction with TLR2.. J Immunol.

[10] Ripoll F, Deshayes C, Pase S, Laval F, Beretti J (2007). Genomics of glycopeptidolipid biosynthesis in *Mycobacterium abscessus* and *M. chelonae*.. BMC Genomics.

[11] Medjahed H, Reyrat J (2009). Construction of *Mycobacterium abscessus* defined glycopeptidolipid mutants: Comparison of genetic tools.. Appl Environ Micro.

[12] Byrd TF, Lyons CR (1999). Preliminary characterization of a *Mycobacterium abscessus* mutant in human and murine models of infection.. Infect Immun.

[13] Nessar R, Reyrat J, Davidson LB, Byrd TF (2011). Deletion of the *mmpL4b* gene in the *Mycobacterium abscessus* glycopeptidolipid biosynthetic pathway results in loss of surface colonization capability, but enhanced ability to replicate in human macrophages and stimulate their innate immune response.. Microbiology.

[14] Krutzik SR, Modlin RL (2004). The role of Toll-like receptors in combating mycobacteria.. Semin Immunol.

[15] Bals R, Hiemstra PS (2004). Innate immunity in the lung: how epithelial cells fight against respiratory pathogens.. Eur Respir J.

[16] Birchler T, Seibl R, Buchner K, Loeliger S, Seger R (2001). Human Toll-like receptor 2 mediates induction of the antimicrobial peptide human beta-defensin 2 in response to bacterial lipoprotein.. Eur J Immunol.

[17] Greendyke R, Byrd TF (2008). Differential antibiotic susceptibility of *Mycobacterium abscessus* variants in biofilms and macrophages compared to that of planktonic bacteria.. Antimicrob Agents Ch.

[18] Kinnula VL, Yankaskas JR, Chang L, Virtanen I, Linnala A (1994). Primary and immortalized (BEAS 2B) human bronchial epithelial cells have significant antioxidative capacity in vitro.. Am J Resp Cell Mol.

[19] Chmura K, Bai X, Nakamura M, Kandasamy P, McGibney M (2008). Induction of IL-8 by *Mycoplasma* membrane in BEAS-2B cells.. Am J Physiol - Lung C.

[20] Livak KJ, Schmittgen TD (2001). Analysis of relative gene expression data using real-time quantitative PCR and the 2^−ΔΔC^T method.. Methods.

[21] Takeuchi O, Kaufmann A, Grote K, Kawai T, Hoshino K (2000). Cutting edge: preferentially the *R*-stereoisomer of the mycoplasmal lipopeptide macrophage-activating lipopeptide-2 activates immune cells through a toll-like receptor 2- and MyD88-dependent signaling pathway.. J Immunol.

[22] Underhill DM, Ozinsky A, Smith KD, Aderem A (1999). Toll-like receptor-2 mediates mycobacteria-induced proinflammatory signaling in macrophages.. Proc Natl Acad Sci USA.

[23] Takeuchi O, Sato S, Ooriuchi T, Hoshino K, Takeda K (2002). Cutting edge: role of toll-like receptor 1 in mediating immune response to microbial lipoproteins.. J Immunol.

[24] Sha Q, Truong-Tran AQ, Plitt JR, Beck LA, Schleimer RP (2004). Activation of airway epithelial cells by toll-like receptor agonists.. Am J Resp Cell Mol.

[25] Wheeler DS, Catravas JD, Odoms K, Denenberg A, Malhotra V (2004). Epigallocatechin-3-gallate, a green tea–derived polyphenol, inhibits IL-1β-dependent proinflammatory signal transduction in cultured respiratory epithelial cells.. J Nutr.

[26] Wang X, Zhang Z, Louboutin J, Moser C, Weiner DJ (2003). Airway epithelia regulate expression of human β-defensin 2 through toll-like receptor 2.. FASEB J.

[27] Harder J, Meyer-Hoffert U, Teran LM, Schwichtenberg L, Bartels J (2000). Mucoid *Pseudomonas aeruginosa*, TNFα, and IL-1β, but not IL-6, induce human β-defensin-2 in respiratory epithelia.. Am J Resp Cell Mol.

[28] Lieber M, Smith B, Szakal A, Nelson-Rees W, Todaro G (1976). A continuous tumor-cell line from a human lung carcinoma with properties of type II alveolar epithelial cells.. Int J Cancer.

[29] Corbiere V, Dirix V, Norrenberg S, Cappello M, Remmelink M (2011). Phenotypic characteristics of human type II alveolar epithelial cells suitable for antigen presentation to T lymphocytes.. Resp Res.

[30] Bermudez LE, Goodman J (1996). *Mycobacterium tuberculosis* invades and replicates within type II alveolar cells.. Infect Immun.

[31] Birkness KA, Deslauriers M, Bartlett JH, White EH, King CH (1999). An in vitro tissue culture bilayer model to examine early events in *Mycobacterium tuberculosis* infection.. Infect Immun.

[32] Garcia-Pereza BE, Mondrago-Flores R, Luna-Herrera J (2003). Internalization of *Mycobacterium tuberculosis* by macropinocytosis in non-phagocytic cells.. Microb Pathog.

[33] Greco E, Santucci MB, Quintiliani G, Papi M, De Spirito M (2009). CpG oligodeoxynucleotides promote phospholipase D dependent phagolysosome maturation and intracellular mycobacterial killing in *M. tuberculosis* infected type II alveolar epithelial cells.. Cell Immunol.

[34] Greco E, Santucci MB, Quintiliani G, Papi M, De Spirito M (2009). CpG oligodeoxynucleotides promote phospholipase D dependent phagolysosome maturation and intracellular mycobacterial killing in *M. tuberculosis* infected type II alveolar epithelial cells.. Cell Immunol.

[35] Rivas-Santiago B, Schwander SK, Sarabia C, Diamond G, Klein-Patel ME (2005). Human β-defensin 2 is expressed and associated with *Mycobacterium tuberculosis* during infection of human alveolar epithelial cells.. Infect Immun.

[36] Kisich KO, Heifets L, Higgins M, Diamond G (2001). Antimycobacterial agent based on mRNA encoding human β-Defensin 2 enables primary macrophages to restrict growth of *Mycobacterium tuberculosis*.. Infect Immun.

[37] Jonsson BE, Gilljam M, Lindblad A, Ridell M, Wold AE, Welinder-Olsson C (2007). Molecular epidemiology of *Mycobacterium abscessus*, with focus on cystic fibrosis.. J Clin Microbiol.

